# Editorial: Extracts from plants and other natural sources: application, characterization, optimization, and their use

**DOI:** 10.3389/fnut.2024.1506537

**Published:** 2024-10-25

**Authors:** Saša D. Đurović, Yulia A. Smyatskaya, Tomislav Tosti

**Affiliations:** ^1^Institute of General and Physical Chemistry, Belgrade, Serbia; ^2^Peter the Great Saint-Petersburg Polytechnic University, Graduate School of Biotechnology and Food Industries, Saint-Petersburg, Russia; ^3^Institute of Chemistry, Technology and Metallurgy - National Institute of the Republic of Serbia, University of Belgrade, Belgrade, Serbia

**Keywords:** extraction, natural compounds, extracts, application, characterization, optimization

Natural compounds have attracted much of the scientific community's attention in the last decades because of a wide range of biological activity. The possibility of changing the synthetic compounds for natural ones in food products was and still is one of the main goals of many studies in this field. For this purpose, different extraction techniques have been developed, followed by the development of many analytical instruments and methods for detecting, identifying, and quantifying isolated natural compounds. Isolation of the natural compounds became a challenging problem of great importance since the natural matrix is a highly complex mixture of different compounds. Besides this, natural compounds, extracts, and their sources are the subject of many studies for their possible application in the food industry and agronomy. Natural compounds are used more often to substitute synthetic antioxidants and other compounds. Moreover, applications in phytotherapy are also very attractive and widely explored these days.

Acquiring the natural compounds from their source implies the application of extraction techniques. Nowadays, these techniques are in two major groups: conventional (maceration, hydrodistillation, and Soxhlet extraction) and nonconventional extraction (ultrasound-assisted, microwave-assisted, subcritical water, supercritical fluid extraction) techniques. The selection of the extraction techniques, conditions, and solvents depends on the nature of the desired compounds. The most recent trends include combining several techniques with different solvents to isolate as many bioactive compounds as possible. Zhu et al. presented in their review article extraction methods for extraction and purification of the polysaccharides from *Perilla fructesens* L. Reviewed approaches are based on the combination of different extraction techniques, conditions, and solvents for isolation of polysaccharides following by multi-step purification to obtain purified products. By contrast, Yan et al. used simple maceration with hot deionized water to isolate pectic polysaccharides from *Veronica peregrina* L., whose extracts were subsequently submitted to purification steps to prepare cleaner products. Optimizing the extraction process is an essential step for achieving the maximal yield of desired compounds. Several mathematical tools are used for this purpose, but response surface methodology (RSM) is the most common one. Kang et al. applied RSM in combination with single-factor experiments and Box-Behnken (BBD) design to optimize the extraction of polysaccharides from red *Panax ginseng* and *Ophiopogon japonicus* waste.

After the isolation and purification of the extracts, the next step is chemical characterization, i.e., the determination of the composition and structures of the isolated compounds and the establishment of qualitative and quantitative profiles of the prepared samples. Many different analytical techniques and methods are used. Chromatographic techniques have become standard techniques for analysis of the extracts and isolated compounds. Shompa et al. reported the application of gas chromatography coupled with mass spectrometry (GC-MS) to evaluate the methanolic extract of the *Baccaurea motleyana* Müll. Arg. seeds, while Shahriar et al. used gas chromatography with tandem mass spectrometry (GC-MS/MS) to evaluate the methanolic leaf extract of *Catharanthus ovalis*. Liquid chromatography is widely used to analyze polyphenolic compounds, which is also confirmed by Ahmed et al., who used the HPLC-DAD technique to evaluate the extracts from *Zingiber roseum* (Roxb.) Roscoe leaf. Moreover, other analytical techniques, such as Fourier transform infrared spectroscopy (FTIR) and nuclear magnetic resonance (NMR), were also reported to be used for this purpose (Yan et al.; Kang et al.).

It is not important only to isolate compounds from their natural sources and to characterize them. It is pretty significant to determine the possible application of obtained extracts and evaluate the biological activity of crude extracts and purified compounds. Numerous techniques and methods have been developed for this purpose, while assessments could be done *in vitro* and *in vivo*. Many spectrophotometric methods have been reported for the evaluation of the antioxidant activity. Among them are the DPPH test, hydroxyl radical scavenging activity, ABTS radical scavenging activity, and many others (Yan et al.). Other *in vitro* methods for assessment of antimicrobial, cytotoxic activity, thrombolytic activity, and membrane-stabilizing activity were also reported and applied (Shompa et al.; Shahriar et al.). Polyphenolic compounds are considered the leading carriers of different biological activities in plants and are widely studied. Hence, Du et al. reviewed the potential of traditional Chinese medicine flavonoids in the treatment of osteoporosis. *In vivo* experiments include the investigation of living subjects such as laboratory animals. Yanan et al. reported the effects of lotus leaf (*Nelumbo nucifera*) ethanol extract on gut microbes and obesity in high-fat diet-fed rats and investigated the mechanism of action. They found that extracts regulate blood lipids and relieve chronic inflammation. Choi et al. reported a protocol for a randomized controlled trial evaluating the effect of *Hibiscus syriacus* L. flower extract on sleep quality, evaluating its effect on human subjects. Besides *in vitro* and *in vivo* studies, the rapid development of information technologies opened the door for computational studies of molecules and prediction of their action. Molecular docking studies become significant tools for evaluating compounds' activity, binding energy for investigated enzymes, and other properties. This method relies on applying existing data about the enzyme structures and structures of natural and synthetic molecules. Using different physico-chemical models and computational simulations, programs predict interaction between investigated structures. Ahmed et al., Shompa et al., and Shahriar et al. used different programs to evaluate the interaction between small molecules of interest and enzymes whose structures were collected from an existing protein data bank.

The quality of the articles presented in this Research Topic illustrates the significance and the value of the subject of this Research Topic: *Extracts from plants and other natural sources: application, characterization, optimization, and their use*. The diversity of the article types, methods, starting materials, and approaches demonstrated the importance of studying compounds from natural sources and the possibility of their wide application in the food, pharmaceutical, and cosmetics industries. The presented articles also showed the necessity of further studies to fill existing gaps and expand our knowledge, which still needs to be completed. This fact opens the door for new studies and Research Topics to overcome these problems and answer all challenges.

